# A medical student's perspective on improving confidence in assessment and management of mental health conditions in UK foundation training

**DOI:** 10.1192/bjb.2024.83

**Published:** 2024-10

**Authors:** Diana Stamatopoulos

**Affiliations:** Medical student, Edinburgh Medical School, University of Edinburgh, Edinburgh, UK. Email: d.stamatopoulos@sms.ed.ac.uk

Gillett et al have conducted a study which demonstrates that newly qualified doctors reportedly feel less confident in their mental health clinical skills compared to managing physical health conditions. This was particularly evident in prescribing psychotropic medications and managing agitation and delirium.^1^ This study suggests that not every junior doctor will acquire these skills during their foundation training, emphasising the importance of developing competency in psychiatry skills during medical school. As a medical student who is approaching their final year of training in the UK, I wish to offer my perspective on the integration of mental health training in the medical school curriculum, as well as identifying potential areas for future research.

Mental health is a theme that will present in various specialties. Therefore, it is required that junior doctors are confident in their clinical skills. The authors suggest that increasing exposure to psychiatry in the medical school curriculum and increasing simulation-based learning during this time can be particularly beneficial to the confidence of newly qualified doctors.^1^ As a medical student who has already undergone teaching and clinical placements in psychiatry, I agree with this conclusion. Through both my placements and teaching, I have been able to involve myself in opportunities to further practise my clinical skills in psychiatry. However, these experiences can be variable depending on the student or placement; therefore, a more uniform method of practice may be required to consolidate students’ learning.

The use of simulated patients has become increasingly common across medical school and acts as a pillar in medical education. From my experiences in training, I have found simulated patient consultations to be very helpful in developing confidence in prescribing and managing acute clinical scenarios. I believe that integrating more psychiatry-related presentations into simulation-based learning during medical school could help increase the confidence of newly qualified doctors in their mental health clinical skills. One study that evaluated simulated learning for medical students further supports this argument; students involved in this study reported that simulated patient consultations allowed them to practise ‘challenging consultations in a safe way’.^2^ The students reported that this was relevant to actual clinical practice, particularly for consultations related to psychiatry. Students also reported that these consultations were realistic, based on what they had encountered during their placements. After practising clinical skills in a supervised environment with constructive feedback, students in this study reported feeling more confident and prepared for working as a doctor.^2^ As a result of these conclusions and the evident benefits of simulation-based learning in medical school, I agree with Gillett et al that more research should be carried out in this area, with a particular focus on mental health training in medical schools. Implementing more simulation-based learning for medical students to practise prescribing psychotropic agents and managing acute mental health presentations could be a way of increasing the confidence of newly qualified doctors in these skills. I appreciate the authors for identifying this limitation among newly qualified doctors and addressing potential ways to mitigate this.
